# ADHD in children and adolescents: Guideline-based online assessment in the consortium project INTEGRATE-ADHD

**DOI:** 10.25646/12541

**Published:** 2024-09-18

**Authors:** Leila Hetzke, Annalena Berner, Sophia Weyrich, Marcel Romanos, Ann-Kristin Beyer, Robert Schlack, Ulrike Ravens-Sieberer, Anne Kaman, Julian Witte, Cornelia Fiessler, Anna Grau, Anna Horn, Peter Heuschmann, Cordula Riederer, the INTEGRATE-ADHD Study Group, Thomas Jans

**Affiliations:** 1 University Hospital Würzburg, Centre of Mental Health, Department of Child and Adolescent Psychiatry, Psychosomatics and Psychotherapy, Würzburg, Germany; 2 Robert Koch Institute, Department of Epidemiology and Health Monitoring, Berlin, Germany; 3 University Medical Centre Hamburg-Eppendorf, Department of Child and Adolescent Psychiatry, Psychotherapy and Psychosomatics, Research Section ‘Child Public Health’, Hamburg, Germany; 4 Vandage GmbH, Bielefeld, Germany; 5 University of Würzburg, Institute of Clinical Epidemiology and Biometry, Würzburg, Germany; 6 University Hospital Würzburg, Clinical Trial Centre, Würzburg, Germany; 7 University Hospital Würzburg, Institute for Medical Data Sciences, Würzburg, Germany; 8 DAK-Gesundheit, Hamburg, Germany

**Keywords:** ADHD, S3 guideline, Online assessment, Children, Adolescents, Telehealth, Feasibility Studies, Germany

## Abstract

**Background:**

The consortium project INTEGRATE-ADHD compared administrative data on the presence of attention-deficit/hyperactivity disorder (ADHD) in children and adolescents with the results of a parent survey and a comprehensive clinical assessment based on the S3 guideline of the Association of the Scientific Medical Societies in Germany (AWMF). Due to the COVID-19 pandemic, the clinical assessment was carried out online.

**Methods:**

The article describes how a guideline-based clinical assessment of ADHD can be implemented in an online setting. A specially developed diagnostic matrix is presented to illustrate the assessment procedures and the diagnostic decision-making process. The matrix is intended to help the diagnostician to gain an overview of the numerous individual findings that have been collected using different assessment perspectives and methods (e.g. diagnostic interviews, rating scales, performance tests) in order to make a well-founded and transparent diagnostic decision.

**Discussion:**

The consortium project INTEGRATE-ADHD has shown that an online assessment can be implemented in a guideline-compliant manner and allows a valid clinical decision. The diagnostic strategy is discussed with reference to international guidelines and recommendations for online diagnostics (e.g. aspects of feasibility, acceptability and safety of the assessment procedures). The challenges and opportunities of using online assessments in clinical practice are also described.

## 1. Introduction

With a prevalence of around 5 %, attention-deficit/hyperactivity disorder (ADHD) is one of the most prevalent mental disorders in childhood and adolescence [1]. The majority of young people affected by ADHD also have at least one other mental disorder or develop comorbid conditions during the course of their disorder [2]. The associated impairments are far-reaching and include, in addition to the reduced mental and physical wellbeing because of the core symptoms and comorbidities, low academic and occupational performance, and poor social relationships in the family, at school, at work and during leisure time [3]. Approximately half of those affected in childhood and adolescence continue to suffer from ADHD and its consequences into adulthood [4].

Early diagnosis and treatment are crucial to work against this development. To ensure a qualified and evidence-based approach, the relevant guidelines for diagnosis and treatment must be taken into account [3]. Precise, guideline-based case definitions are also important in the context of health care research. They allow the evaluation of existing diagnostic and treatment capacities and thereby the evidence-based formulation of recommendations for their improvement. This is the aim of the consortium project INTEGRATE-ADHD, which is funded by the German Innovation Fund of the German Federal Joint Committee [5, 6]. A randomly selected sub-sample (*n* = 202) from the group of children and adolescents whose parents participated in the INTEGRATE-ADHD online survey (Infobox) underwent a comprehensive clinical assessment according to the German S3 guideline on ADHD of the Association of the Scientific Medical Societies in Germany (Arbeitsgemeinschaft der Wissenschaftlichen Medizinischen Fachgesellschaften, AWMF) [3]. Inclusion and exclusion criteria, recruitment procedure and sample characteristics of the study are described elsewhere [6].

**ADHD in Germany – Comparison and integration of administrative and epidemiological ADHD diagnostic data through clinical assessment (INTEGRATE-ADHD)**

**Consortium partners:** Robert Koch Institute Berlin, Department of Epidemiology and Health Monitoring, Germany; University Hospital Würzburg, Department of Child and Adolescent Psychiatry, Psychosomatics and Psychotherapy, Germany; University Medical Centre Hamburg-Eppendorf, Department of Child and Adolescent Psychiatry, Psychotherapy and Psychosomatics, Research Section ‘Child Public Health’, Germany; Vandage GmbH, Germany; University of Würzburg, Germany, Institute for Clinical Epidemiology and Biometry, Germany; DAK-Gesundheit, Germany

**Data holder:** Robert Koch Institute

**Objectives:** Identification of potential causes for the discrepancies between administrative ADHD diagnostic data (based on health insurance claims data) and epidemiological ADHD diagnostic data (based on surveys) for Germany, integration and validation of these data through a guideline-based clinical examination

**Study design:** Cross-sectional online survey, additional clinical examination of a sub-sample, data linkage with administrative health insurance data

**Population:** Children and adolescents who were insured with DAK-Gesundheit in 2020 and who were 0 to 17 years old at that time and for whom an administrative ADHD diagnosis labelled as confirmed was available in at least one quarter

**Gross sample**: 24,880 children and adolescents insured with DAK-Gesundheit with an administrative ADHD diagnosis

**Net sample:** 5,461 surveyed parents, 202 clinically examined children and adolescents

**Data collection period:** October 2021 to August 2022 (online survey), January 2022 to January 2023 (online clinical examination)

More information in German at www.rki.de/integrate-adhd

Due to the COVID-19 pandemic, the clinical assessment was carried out online via video chat. The aim of this article is to describe in detail the diagnostic procedures applied. On the one hand, this is intended to make the methodology used in INTEGRATE-ADHD transparent. On the other hand, it will show how a complex collection of diagnostic information can be carried out in a structured way within the framework of online diagnostics. This collection of information includes the recording of symptoms and the level of functioning of the child or adolescent in different areas of life over time, as well as the recording of differential diagnostic findings. All relevant information contributes to a final clinical diagnostic decision. A diagnostic matrix has been developed for this purpose, which can also help diagnosticians in clinical practice to gain an overview of the individual findings and thus facilitate diagnostic decision-making. In addition, the diagnostic matrix can be used to describe the diagnostic decision-making process.

Finally, this article describes the implementation of a guideline-based assessment of ADHD in an online setting. This was essential during the COVID-19 pandemic, when contact restrictions to contain transmission routes made face-to-face testing difficult. In the future, online assessment can also be an important adjunct to on-site examinations, especially when access to specialists is limited due to low coverage or lack of mobility of families. By avoiding travel and potential delays, it is more time-saving, cost-effective and sustainable than on-site assessment for both families and diagnosticians. The preconditions and possibilities of online diagnostics of ADHD have recently been discussed, not least due to the pandemic [7–9]. Our project aims to contribute to this discussion.

In the following, the basic features of ADHD diagnostics according to the German S3 guideline are presented, before the online assessment carried out in the project is described and discussed in detail.

## 2. Online assessment of ADHD in the INTEGRATE-ADHD project

### 2.1 Diagnosis according to the S3 guideline

ADHD is characterised by the presence of the core symptoms of inattention, hyperactivity and impulsivity. Diagnosis requires that the symptoms

1. onset is during preschool or primary school years (before the age of six according to the World Health Organization’s International Statistical Classification of Diseases and Related Health Problems (ICD-10) [10] or before the age of twelve according to the American Psychiatric Association’s Diagnostic and Statistical Manual of Mental Disorders (DSM-5) [11]),
2. are inconsistent with the developmental stage of the individual,
3. do not occur for only a short period of time (a few months), but for a longer period of time (at least six months),
4. cause significant distress or impairment and occur in more than one area of life (e.g., peer relationships, family life, school or work – so-called pervasiveness of symptoms), and
5. cannot be better explained by other mental disorders.

ADHD is a risk factor for developing other mental disorders which need to be considered when diagnosing. The full picture of ADHD (the presence of inattention as well as hyperactivity and impulsivity) is called ‘disturbance of activity and attention’ in ICD-10. In DSM-5, in addition to this combined presentation of ADHD, two presentations with predominant inattention and with predominant hyperactivity and impulsivity are further specified. The diagnostic criteria in ICD-10 and DSM-5 are very similar, but differ in the required number of symptoms of hyperactivity and impulsivity, as well as in the age of onset and subtype classification. It is therefore possible that the diagnostic decision on the presence of ADHD may differ depending on whether ICD-10 or DSM-5 is used. The diagnostic criteria for ADHD in ICD-11 [12], which will be used after an as yet unspecified transition period, are largely congruent with those in DSM-5, so the diagnostic procedure described in this article will continue to be valid.

According to the German AWMF S3 guideline [3], the diagnostic strategy should be multimodal, i.e. information should be obtained from different informants (affected individuals and important others, e.g. parents and teachers) using a variety of methods (interviews, standardised self and proxy rating scales, behavioural observation, psychological tests and medical examinations, review of written reports and school reports), taking into account the development of symptoms over time in different settings.

The clinical interview should focus on assessing

1. current ADHD symptoms and their situational variability (type, frequency and severity) in different areas of life,
2. the resulting functional impairment,
3. co-existing disorders,
4. family history (family situation, resources and stressors),
5. the patient’s medical history, focusing on the development of symptoms in the context of general development and pre-treatment, as well as for further therapy planning on
6. the resources, wishes and needs of the patient and her or his caregivers.

In addition, an observation of the patient’s behaviour during the assessment, a mental state examination and a physical-neurological examination with an evaluation of the developmental status should be carried out. In addition to proxy reports, the patient’s self-report becomes more important as the patient grows older. The use of questionnaires is also recommended. Psychological tests of intelligence and executive function (goal-directed action planning, impulse control, selective attention, or working memory) may also be helpful. Routine testing of laboratory parameters is not necessary, but may be indicated, as may device-based examinations such as electroencephalography (EEG), if there are relevant medical indications for differential diagnosis. The AWMF S3 guideline provides information on which conditions should be considered as a differential diagnosis and on the evaluation of possible comorbidities. After reviewing all relevant information, the clinical expert makes an integrated clinical evaluation and decides on the presence or absence of an ADHD diagnosis. This diagnostic strategy has been implemented in the project and is detailed below.

### 2.2 Guideline-based clinical assessment in the online setting

#### Diagnostic strategy

**Figure 1:**
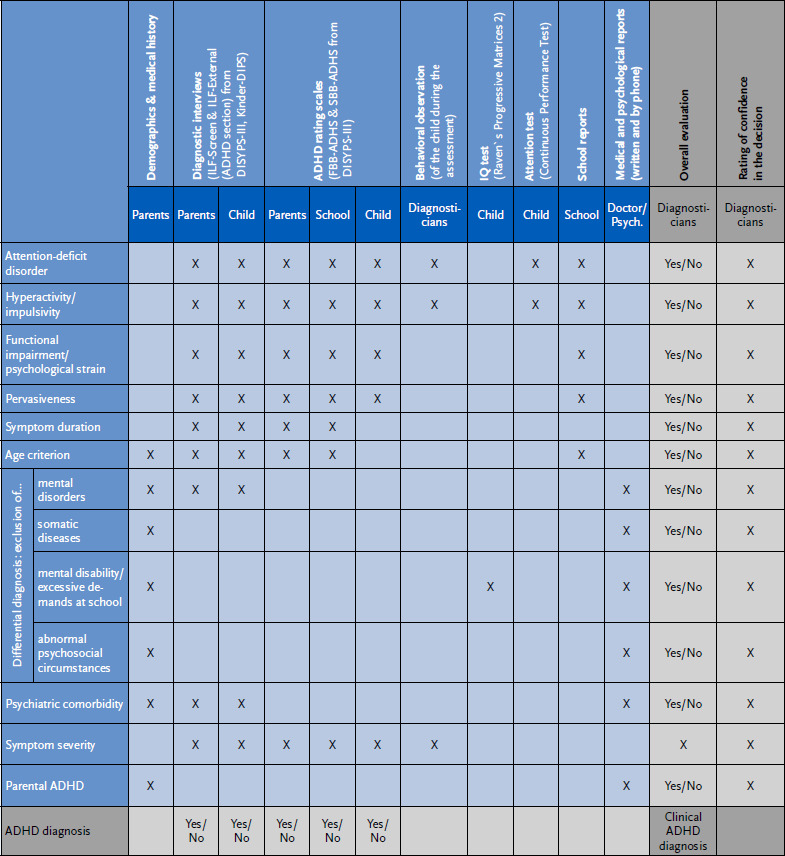
Diagnostic matrix – Overview of the diagnostic strategy. Source: Own depiction ADHD = attention-deficit/hyperactivity disorder, ILF = diagnostic interview (‘Interviewleitfaden’), DIPS = diagnostic interview (‘Diagnostisches Interview bei psychischen Störungen’), FBB = proxy rating (‘Fremdbeurteilungsbogen’), SBB = self rating (‘Selbstbeurteilungsbogen’), DISYPS = Diagnostic System for Mental Disorders (‘Diagnostik-System für Psychische Störungen’), Psych. = psychologist Cells marked with an X illustrate the content for which diagnostic information was collected using which assessment method or source. For reasons of overview, the matrix has been reduced somewhat (the version used contains separate columns for each of the interviews on ADHD and other disorders).

The diagnostic matrix in Figure 1 provides an overview of the clinical diagnostic strategy and diagnostic decision-making process in the INTEGRATE-ADHD project. The rows list the diagnostically relevant content that is captured by the different methods or sources. The columns show first the assessment methods or sources of information and then the corresponding evaluation of the diagnostician. Cells in the diagnostic matrix marked with a cross indicate for which content diagnostic information was obtained using which method or source. This information is summarised in one or more characteristic values (e.g. whether the area is abnormal or not). The rows attention-deficit disorder, hyperactivity/impulsivity, functional impairment/psychological strain, pervasiveness, symptom duration and age criterion together with the following rows for differential diagnosis represent the diagnostic criteria. Psychiatric comorbidities, severity indices and information on parental ADHD follow. The information collected by each assessment method or source (columns of the matrix) is summarised and scored (e.g. whether or not diagnostic criteria are met on the basis of parental information in the interview). On the other hand, the diagnostic information available for each domain or content (rows of the matrix) is also summarised and scored (e.g. whether or not an attention deficit disorder is present, considering all the findings). The diagnostician’s confidence in his/her decision is also recorded. Finally, the diagnostician makes a ‘best estimate’ diagnosis according to ICD-10 and DSM-5 based on the often not fully consistent individual findings. This integrative clinical evaluation of the collected information is a central task of the diagnostician. The clear structuring of the individual diagnostic information in the matrix facilitates the diagnostic decision-making process. In addition, by the scoring of each individual finding this decision-making process can be empirically analysed.

#### Assessment methods and sources

##### Demographics and medical history

In order to identify relevant contextual factors and their role in the development of symptoms, basic demographics, personal history, family history, psychosocial stressors and medical history were collected through a parent interview. Parents completed a pre-screening questionnaire to collect basic information, which was then compared and supplemented in the parent interview. An interview guide was used, which included a screening interview on psychosocial stress (abnormal psychosocial circumstances based on the Multiaxial Classification of Mental Disorders in Childhood and Adolescence, MAS [13]). Checklists on diseases relevant to the differential diagnosis and adverse effects of medication in ADHD-like symptoms were also included.

The interview guide and checklists used to structure the diagnostic process can be requested via the correspondence address.

##### Diagnostic interviews

Semi-structured diagnostic interviews were conducted with a parent and with the children and adolescents themselves from the age of eight.

The German Screening Interview for Mental Disorders (ILF-Screen) from the Diagnostic System for Mental Disorders in Childhood and Adolescence (DISYPS-III) [14, 15] was used as an initial screening. The ILF-Screen asks about central symptoms of the most important disorders. In the event of an abnormal screening result, the relevant sections of the German Diagnostic Interview for Mental Disorders in Childhood and Adolescence (Kinder-DIPS) were administered [16, 17]. The Kinder-DIPS is a German translation of the Anxiety Disorders Interview Schedule – Revised, ADIS-R [18] and has been further developed into an independent, semi-structured clinical interview [16]. Studies on the psychometric properties of previous versions of the Kinder-DIPS show good to very good intercoder-reliability for the parent and child versions as well as satisfactory results for the validity [16, 19, 20]. In addition, a high level of satisfaction and acceptance of the assessment by interviewers and parents and children has been demonstrated [21].

The ADHD domain was assessed for each participant using the ADHD section of the German Clinical Interview for Externalizing Disorders (ILF-EXTERNAL) from the DISYPS-III [14, 15]. The ILF interviews are very detailed, disorder-specific, semi-structured interviews that provide definitions, clinical descriptions, example questions and examples of severity classifications for each symptom criterion of the disorder to be assessed. Diagnostic criteria are recorded according to the ICD-10 and DSM-5 classification systems. Initial studies in children have shown good inter-rater agreement and satisfactory to good internal consistencies for the ILF-EXTERNAL and its symptom scales. Validity studies show high correlations between the scales of the ILF-EXTERNAL and parent rating scales from the DISYPS-III and satisfactory correlations with the corresponding scales of the Child Behaviour Checklist, CBCL/6-18R [15]. The ILF-EXTERNAL is recommended as an instrument in the German AWMF S3 guideline for ADHD [3]. For a psychometric evaluation of the ILF-EXTERNAL within the INTEGRATE-ADHD project see Weyrich et al. [22].

##### Rating scales

In addition, proxy ratings (FBB = Fremdbeurteilungsbogen) of ADHD symptoms by a parent and a teacher or preschool educator (FBB-ADHS or FBB-ADHS-V for preschool age) and self-ratings (SBB = Selbstbeurteilungsbogen) of children and adolescents aged eleven years and older (SBB-ADHS) from the DISYPS-III diagnostic system [14] were administered. The selection of rating scales follows the recommendations of the German AWMF S3 guideline [3]. The rating scales are based on the diagnostic criteria of the ICD-10 or DSM-5 and are very similar in structure to internationally established scales such as the Swanson, Nolan and Pelham-IV (SNAP-IV) scales [23]. Several studies have shown satisfactory to very good internal consistencies for the different versions of the FBB-ADHS(-V) and the SBB-ADHS [14, 24, 25]. In a study of teacher ratings, Breuer et al. [24] also found sufficient to high interrater reliability (agreement between scores obtained by different raters) and good test-retest reliability (agreement between scores obtained at repeated measurements). Validity findings are also available [24].

The dimensional scoring of the ILF-EXTERNAL scales and the proxy and self-rating scales was not norm-referenced in our project as there are no comparable representative standardisations across the instruments. Coghill et al. [26] and Coghill et al. [27] provide recommendation on the interpretation of the ‘average rating of items’ (ARI) scores. In accordance with the DSM-5 requirement of at least six items with a score ≥ 2 in a symptom domain (out of a total of nine items, each item score ranging from 0 to 3), we considered an ARI score ≥ 1.33 as ‘elevated’ and a score ≥ 1.8 as ‘clearly elevated’. These scores are intended as a guide for interpretation only and should not be taken as obligatory cut-off scores. In addition to the dimensional scores, we used a dichotomous scoring for the rating scales and the ILF-EXTERNAL scales as to whether or not the minimum number of items with elevated scores required for a diagnosis was met.

##### Intelligence diagnostics

In accordance with the recommendation of the AWMF S3 guideline on ADHD, an orienting assessment of intelligence was carried out to get a basis to differentiate ADHD symptoms from externalising symptoms related to low intelligence or excessive demands at school. Children and adolescents from the age of four were assessed using the digital short version of the Raven’s Progressive Matrices 2 (Raven’s 2) [28]. The Progressive Matrices tests are internationally established measures of general intelligence, of which the Raven’s 2 is a new edition with an international norm sample. The short version consists of 24 items and takes approximately 20 to 30 minutes for administration including the instruction. A study conducted in the US found good overall reliability for the digital short form [28]. In addition, high correlations were found between the Raven’s 2 and previous versions of the Raven’s test, so that the validity of the Raven’s series established for intelligence diagnostics can be assumed to be also given for the Raven’s 2. In the INTEGRATE-ADHD project, IQ scores were defined that should provide clear indications of a mental retardation (IQ < 70) or excessive demands depending on the type of school attended by the child (German Grundschule and Mittelschule: IQ < 80, Realschule and Gymnasium: IQ < 90).

##### Executive functions

The Continuous Performance Test, CPT [29], was administered from the age of four. The CPT is an internationally established test that takes approximately 30 minutes to administer. It measures selective attention, sustained attention and aspects of cognitive impulsivity or response inhibition and working memory in the OX paradigm. The letters H, O, T, X or Z are presented for 200 milliseconds at two-second intervals. The subject’s task is to respond as quickly as possible to the letter X by pressing a button if the letter O was presented previously. With regard to the psychometric properties of the test, only calculations on similar versions of the CPT applied are available. Acceptable retest reliability and acceptable to excellent internal consistency were found, as were correlation coefficients on a medium level for validity [29]. In our clinical assessment, attention performance was considered to be abnormal if the mean reaction time, its standard deviation or the number of omission errors (omission error: no reaction to the test stimulus although a reaction should have occurred) was below average (deviation from the mean of the norm sample: z ≥ 1). To measure impulse control, the number of commission errors (also called confusion or false alarm errors: responding to a test stimulus that should not have been responded to) was used in the same way [29].

##### Behavioural observation

The behaviour of the children and adolescents during the clinical assessment was rated by the clinician using the observation scale ‘Symptom level during the assessment’ (part of the DISYPS-III [14]) (four-point scale from 0 to 3). Scores ≥ 2 in the area of inattention or in the area of hyperactivity or impulsivity were considered abnormal.

##### Severity rating

Severity of overall symptoms, including possible comorbid conditions, was rated by the clinician using the Clinical Global Impressions severity scale, CGI-S [30]. The rating was based on the overall information gathered by the end of the clinical assessment. A CGI-S score > 3 was considered severe.

##### External findings, School reports

Although the style of school reports differs from country to country, they often contain detailed descriptions of the child’s behaviour in the school situation, especially during the primary school years. For schoolchildren, all report cards from grades 1 to 5, if available, and for older pupils the current report cards (last annual and interim reports) were evaluated by the diagnostician using an instruction. The extent to which the core symptoms of ADHD were described in the available reports was recorded for each of the school years 1 to 5 and for the most recent year. The focus on symptoms in grades 1 to 5 takes into account the age criterion of the DSM-5, according to which the diagnosis requires the onset of symptoms before the age of twelve years. Indications of possible excessive demands in terms of school performance were also recorded.

##### Medical findings, therapeutic findings, pediatric preventive check-ups

Medical and therapeutic findings, including the paediatric health record (in German ‘U-Heft’), were reviewed and assessed by the diagnostician with regard to the child’s development, important differential diagnoses and comorbidities, psychosocial stressors, and evidence of parental ADHD. In addition, where possible, a telephone interview was conducted with the person providing medical or psychotherapeutic treatment. These external findings were important for the collection of additional differential diagnostic information.

#### Examiner training and supervision

The clinical assessments were carried out by one of seven psychologists or psychotherapists in education at the Department of Child and Adolescent Psychiatry, Psychosomatics and Psychotherapy at the University Hospital of Würzburg. They received special training in the use of the diagnostic interviews (two half days for the ADHD section of the ILF-EXTERNAL, one half day for the Kinder-DIPS). For the ADHD section of the ILF-EXTERNAL, two video ratings took place following the training sessions, with the examiner ratings being compared with an expert rating. A standard of agreement with the expert rating had to be met at least in the second rating (deviation of the sum scores of the ADHD scale < 3 points). The diagnostic instruments were also practised as part of routine examinations in the clinic’s ADHD outpatient service. Weekly consultation and coordination meetings with the project management took place during the preparation and data collection phases of the study. Each diagnostic assessment in the project was supervised by medical and psychological experts.

The interrater agreement (agreement between the findings of different diagnosticians) of the central measure of ADHD symptoms (parent interview ILF-EXTERNAL) was determined by re-coding the video recordings of the interview in a randomly selected sub-sample of 65 participants and was found to be very good (Cohen’s kappa, intraclass correlation ICC) [22]).

#### Implementation of guideline-based clinical assessment

**Figure 2:**
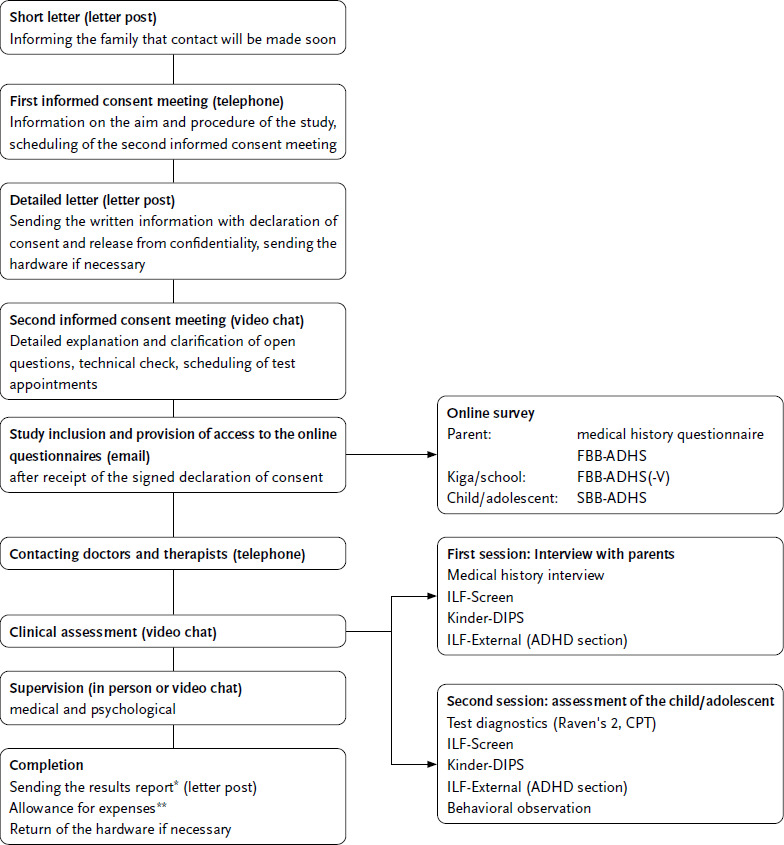
Workflow of the assessment procedure. Source: Own depiction FBB = proxy rating (‘Fremdbeurteilungsbogen’), SBB = self rating (‘Selbstbeurteilungsbogen’), ADHD= attention-deficit/hyperactivity disorder, Kiga = kindergarden, ILF = diagnostic interview (‘Interviewleitfaden’), DIPS = diagnostic interview (‘Diagnostisches Interview bei psychischen Störungen’), Raven’s 2 = Raven’s Progressive Matrices 2, CPT = Continuous Performance Test. ^*^In the results report for the parents, individual recommendations for further treatment or further diagnostics were given if indicated. ^**^The expense allowance amounted to 200 euros per family.

The flowchart in Figure 2 provides an overview of the assessment procedure. The multi-stage process of contacting, informing, diagnosing and reporting results involved written communication by post, email and online portals, and verbal communication by telephone and video chat.

Clinical assessments were conducted via video chat (Skype for Business, S4B). If parents and children/adolescents agreed, the chat was recorded for use in supervisory sessions and for secondary analyses to determine inter-rater reliability. The clinical assessment was structured using an anamnesis guide, checklists and interview documentation forms. Results were documented after the diagnostic appointments in the REDCap web application. REDCap is a research electronic data capture (EDC) system hosted on the University of Würzburg server. The questionnaires were also administered via REDCap with the permission of the copyright holder (Hogrefe). Participants accessed their questionnaire via a link after entering an individual identification code. The scoring of the ADHD rating scales was done automatically via REDCap, as was the generation of summarised overviews of the available diagnostic information. The test application was also browser-based. To complete the CPT, a link was sent to the family, which the child or adolescent used to log into Hogrefe’s HTS5 testing platform, with parental assistance if necessary. The screen was shared with the examiner during the test via S4B. To administer the digital short version of Raven’s 2, the examiner logged in via the Pearson platform ‘Q-global’ and presented the items to the child or adolescent on the shared screen via S4B. The answers were entered by the examiner. This data entry was not critical, as Raven’s 2 is a so-called ‘power test’, in which, unlike a ‘speed test’, the speed of answering is not important.

The clinical assessment was carried out in two sessions of approximately three to four hours each, with the first session usually focusing on the medical history and the interview with the parents, and the second session on the psychological tests and the interview with the child. Appointments with children and adolescents allowed for breaks and spontaneous interruptions as needed. If necessary, they could be split into two shorter appointments.

## 3. Discussion

This article describes how the INTEGRATE-ADHD project carried out a guideline-based clinical ADHD assessment in an online format. The online format became necessary due to contact restrictions during the COVID-19 pandemic. A faceto-face diagnostic was originally planned. However, it was shown that online assessment could be implemented in line with the guidelines. The reliability of the online parent interviews was also ensured by a second coding of the video recordings of a randomly selected sample of interviews. This telemedicine approach, initially born out of necessity, resulted in a clear benefit. In order to provide suggestions for the use of this online assessment of ADHD in research and clinical practice, aspects of the feasibility, acceptability and safety of the diagnostic procedure are considered below. Alternatives to the diagnostic procedures we used are also presented, and general recommendations for online diagnostics are given, outlining their opportunities and limitations. Finally, we summarise the benefits of the diagnostic matrix developed in the project for structuring the collection of diagnostic information.

The offer of an online assessment was well accepted by the families. Of the 431 families randomly selected and contacted for participation, only two refused clinical diagnosis via video chat with accompanying video recording [6]. Only one family was unable to use the technical equipment. Overall, the online procedures were technically easy to implement. Difficulties, such as connection problems, were overcome by planning ahead in the form of trial runs. Adjustments to the test procedure were easy to make, for example if a child needed a break or if parental support was needed. Only in one of the 202 children or adolescents tested did the results not provide enough information to make a sufficiently reliable diagnostic decision about the presence of ADHD. However, there was no systematic survey of the participants at the end of the assessments to evaluate their satisfaction with the assessment procedures.

Other studies have also demonstrated good feasibility and acceptance of online assessments. For example, Elford et al. [31] reported on cross-disorder telepsychiatric clinical examinations via video chat in 4 to 16 year old children and adolescents and compared the results with those obtained in face-to-face examinations. They found that online diagnoses were consistent with face-to-face diagnoses in 96 % of cases, with no differences in the examiners’ subjective confidence in their diagnostic decision. However, examiners showed a preference for face-to-face examinations, while parents showed no clear preference for either setting and appreciated the benefits of not having to travel long distances for online diagnosis. Nelson et al. [32] showed in a pilot study that the American Academy of Pediatrics guidelines were well adhered to when diagnosing ADHD in a telemedicine setting. There are also positive reports on online assessments for other disorders (see for example [33–35]). However, in an international comparison, no comprehensive, guideline-conform clinical ADHD assessment in an online setting, as was the case in the INTEGRATE-ADHD project, has yet been described. The project shows for the first time that diagnostic appointments lasting several hours over two days can be conducted online. The experience of children and families with distance learning during the pandemic, where families had acquired both the technical equipment and the skills to use it, certainly contributed to the successful implementation. Our project also demonstrated a high reliability of online diagnoses [22].

The European ADHD Guidelines Group requires that an online diagnosis of ADHD is based on the standards of face-to-face diagnosis and is in accordance with the guidelines [7]. Our online clinical assessment takes this into account and is based on the German AWMF S3 guideline. In terms of diagnostic standards, the German guidelines do not differ significantly from other internationally established guidelines [36–39]. Therefore, our approach is also of international relevance.

With regard to the choice of assessment instruments, alternatives to our approach can be discussed. The DISYPS-III rating scales that we used have the advantage of incorporating the current ICD-10 and DSM-5 criteria. They also offer good international comparability, as the widely used SNAP scales are almost identical in structure and scaling [40]. Alternatively, the Conners-3 scales [41] could be used. The semi-structured ADHD interview ILF-EXTERNAL that we used is highly differentiated. It is therefore time-consuming and represents the most comprehensive procedure in German-speaking countries, especially as the Kiddie Schedule for Affective Disorders and Schizophrenia (Kiddie-SADS) is not adapted to the DSM-5 criteria [42, 43]. To save time, the Kinder-DIPS was used for differential diagnosis and assessment of comorbid mental disorders. The DIPS is so short that its administration is equivalent to a free, checklist-oriented exploration.

However, due to time constraints, we believe that a diagnostic checklist-based approach to the assessment of ADHD in clinical practice is feasible, provided that it can be administered by experienced clinicians. The selection of appropriate online tests to measure intelligence and executive functions in children and adolescents has been challenging. Good alternatives are not available on the standard online platforms of test publishers for German-speaking countries. There is a clear need for development. After all, the digital performance tests are not designed to be completed independently by the child or adolescent and require professional instruction and guidance from the diagnostician. We have solved this in our online diagnostics by accompanying the test administration in a video chat. Overall, the use of instruments offered via the platforms of test publishers is highly recommended for online diagnostics. In-house programming, e.g. via REDCap, as we did for the rating scales, is demanding as it requires complex consultations and agreements with the publishers regarding copyrights and invoicing. In addition, in-house development is limited to the collection of raw data. Further automated electronic processing of data in clinical practice in the healthcare sector would require certification under the Medical Devices Act. This is unlikely to be affordable for individual users. There are few areas of ADHD diagnosis that cannot be carried out online. These include the physical-neurological examination and, if necessary, additional instrumental or laboratory tests in some cases. However, for differential diagnostic aspects, external reports can be considered in conjunction with the results of online diagnostics [7]. If necessary, additional on-site investigations can be initiated from the online setting.

**Figure 3:**
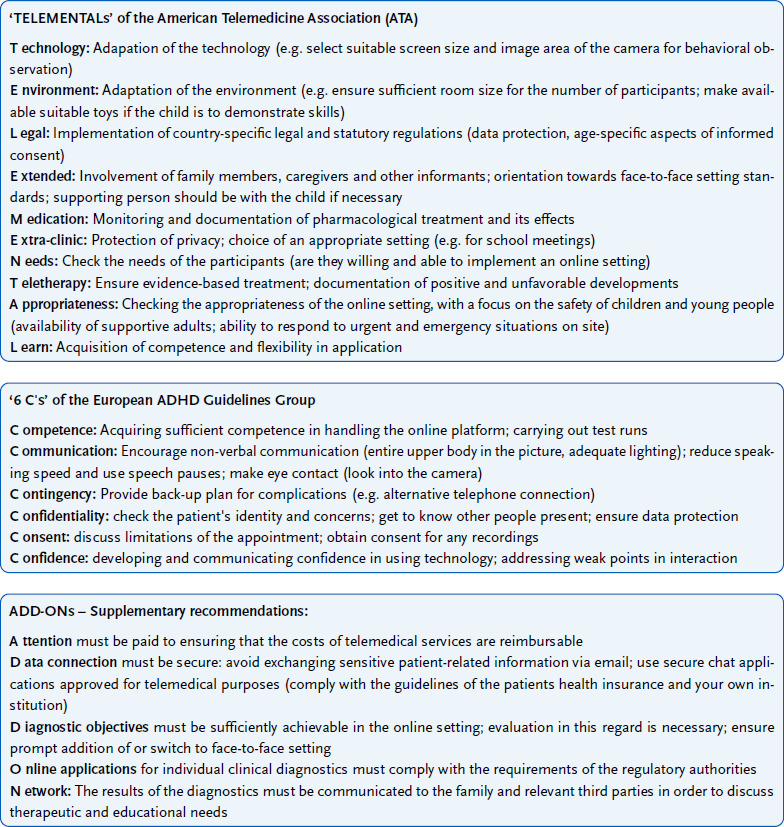
Summary of the guidelines and recommendations from the American Telemedicine Association and the European ADHD Guidelines Group for telemedicine diagnosis and treatment with supplements. Source: American Telemedicine Association [44], European ADHD Guidelines Group [7] ADHD = attention-deficit/hyperactivity disorder

When conducting online diagnostics, it is important to follow key principles of quality assurance and the protection of patients’ rights and privacy. These principles have been published by various organisations and associations. For example, the American Telemedicine Association (ATA) [44] has published guidelines for the use of telemedicine for diagnosis and treatment of children and adolescents via video chat. The six C’s (competence, communication, contingency, confidentiality, consent, confidence) of the European ADHD Guidelines Group provide guidance specifically for online diagnosis [7]. Figure 3 summarises these recommendations with additions from the experience of this project. In particular, the protection of privacy by the selection of secure platforms and applications should be emphasised.

The benefits and opportunities of online diagnostics lie in its flexibility of time and place. This saves time for everyone involved, leading to shorter waiting times, a wider range of appointments, better compatibility with school, work or family commitments, and cost savings. People with physical impairments can be spared unnecessary stress. Reducing the spread of infection is an additional benefit. Online questionnaires can be completed flexibly and close to the symptoms in everyday life. Last but not least, reliable online diagnostics are an important care option for rural or underserved areas. The online setting can also be particularly attractive to children with ADHD if they have an affinity for media. In addition, the greater distance from the examiner associated with the online setting may have a positive effect on some participants’ willingness to open up. Digitalisation and automated data analysis are also advantageous in terms of minimising assessment errors and reducing assessment time. In our project, the online approach was related to the diagnosis of ADHD. However, it can also be used for economic evaluation of therapy. Also worth mentioning in the context of online diagnostics are the possibilities for timely recording of symptoms in direct relation to everyday life through regular short assessments via mobile devices (ecological momentary assessment) [45]. There are also significantly more publications on online therapy for ADHD than on diagnostics [46, 47]. This suggests that there is considerable potential for telemedicine to expand care for children and adolescents with ADHD.

However, there are also limitations to online diagnosis. Some barriers to the use of telemedicine approaches relate to technical aspects such as the availability of a stable internet connection, other technical requirements (e.g. PC or laptop with keyboard and mouse, as some tests cannot be performed on a tablet or smartphone) and the user skills of the participants. Other factors include additional organisational effort, such as sending documents by post (e.g. certificates), limitations in the case of speech and other communication disorders, possible lack of familiarity and willingness to open up due to the distance to the clinician in virtual contact, possible inappropriate influence of assisting caregivers, limited possibilities for observing behaviour, limitations in clinical diagnosis of young children and people with intellectual disabilities, as well as distance-related difficulties in recognising moments of danger for children and adolescents with regard to neglect, violence and abuse. The ability to respond to acute crisis is also limited in online settings. It is therefore crucial to identify barriers to use early, to offer additional face-to-face appointments or, in certain cases, to dispense with online diagnostics completely (on the opportunities and barriers of online diagnostics, see also [48] or [7]). Ultimately, online and face-to-face settings are not mutually exclusive. Alternating and hybrid models can be implemented. The choice of setting should also be based on the preferences of the child and family.

In addition to these telemedical aspects, we have shown in our article how to structure a guideline-compatible clinical diagnosis of ADHD using the diagnostic matrix presented. The diagnostic matrix provides the clinician with a good overview of complex diagnostic information, making clinical decision easier and more transparent. The overview of the findings also makes it easier to inform the individual and their family about the results of the assessment and to supervise diagnosticians in training. The general structure of the matrix is easily transferable to the diagnosis of other mental disorders. However, the diagnostic matrix does not provide an algorithm for how inconsistent individual diagnostic findings should be weighted when making a diagnostic decision, e.g. when parents and teachers report abnormal findings but self-reports and behavioural observations indicate the opposite. There is no reliable empirical basis for an algorithm. The integration of findings in terms of a ‘best estimate diagnosis’ remains the central task of the diagnostician. The INTEGRATE-ADHD project will investigate how the various individual diagnostic findings correlate and which ones are the main determinants of the diagnostician’s decision.

## Key statement

- A guideline-based clinical assessment of ADHD can be carried out online with children and adolescents and their carers using video chat and testing platforms.
- Necessary differential diagnostic information can be obtained from previous therapists, so that an additional face-to-face assessment is usually not necessary.
- The online assessment was well accepted by the participants, and has been able to ensure a high level of reliability of the data collected online via parent interviews.
- A challenge of online assessment is to ensure confidentiality and data protection.
- Online assessment offers an alternative to face-to-face diagnostics in cases of difficult accessibility or contact restrictions, to save costs and time, or for reasons of sustainability.
